# Applicability of Magnetic Sensors in Interlocking Systems

**DOI:** 10.3390/s22124314

**Published:** 2022-06-07

**Authors:** Răzvan Andrei Gheorghiu, Florin Bădău, Valentin Iordache

**Affiliations:** Transport Faculty, POLITEHNICA University of Bucharest, 060042 Bucharest, Romania; florin.badau@upb.ro (F.B.); valentin.iordache@upb.ro (V.I.)

**Keywords:** interlocking, relay monitoring, magnetic sensors, rail systems, rail reliability

## Abstract

Rail interlocking systems follow the progress of technology and train speeds. Nowadays, new systems are designed based on microcontrollers and reliable software, requiring many hours of testing to ensure their proper functionality and bug-free coding. However, in many countries, there are still older relay solutions implemented that are not envisaged to be upgraded in the near future partially due to costs but mainly due to the fact they function very well, being based on a highly reliable element: the relay. However, specialized maintenance personnel are becoming more difficult to find, so automation systems that check the proper relay functionality are a key element to ensure a longer lifetime of existing systems. In this article, the authors present a monitoring solution based on magnetic sensors that proved capable of provide reliable information about the relays, hence revealing the first step in a relay automated control system.

## 1. Introduction

From an outsider’s perspective, the railway system may not seem that complicated at the first glance: just trains running on tracks, with stations laid alongside them. The only notable elements of the system, besides the trains themselves, are the occasional light signal and branching of the lines. The most important elements of the railway system are hidden from the eyes of the public and work in the background to ensure the safety of all involved parties (passengers, railway staff, and bystanders) [1].

The routing of trains inside the limits of train stations is regulated by the interlocking system. This is connected to all outside equipment (rail signals, point switches, derailers, track circuits, axel counters, and so on) and is responsible for their proper functioning. It also ensures that all commands received from the station operator are processed in such a manner that all safety conditions are met.

The first interlockings were designed over 150 years ago, not long after the rapid expansion of the railway network in Europe and North America [2]. With the progress of technology, interlockings have also changed over this long period of time. The first generation of railway interlockings operated on a pure mechanical logic with points and signals commanded and controlled by a system of levers, wires, and pulleys [3]. The railway logic was also implemented mechanically, with a system of interlocking rods preventing the operator from performing certain actions when the safety conditions were not met [4].

The next step in the evolution of these systems came with the expansion of the electrical distribution network [3]. Relays became the fundamental component around which new interlockings were designed and built. Given the binary nature of relays, which can either be picked or dropped, the logic of relay interlockings is governed by the rules of Boolean algebra [5]. By wiring the coils and contacts of the relays in specific configurations, advanced logical functions may be designed and implemented [4,6]. These properties allowed for the operation of more complex stations at higher capacity.

Today’s state of the art interlocking systems are electronic, with software logic playing the central role in ensuring railway operations are carried out safely. The advantages of electronic interlockings are linked to a higher degree of flexibility of the system, the ability to operate multiple stations from the same control point and easier maintenance [7,8]. Electronic interlockings can also be easily integrated with the latest railway signaling systems, such as the European Train Control System (ETCS). The reliability of electronic interlockings is ensured by established hardware and software redundancy architectures: 2002, 2003, or 2∙2002 [4].

However, we have to consider that even at the present time, many national railway networks, especially in Europe, still have a large share of their rail stations controlled by legacy interlocking systems (mechanical or relay). While these systems will be eventually replaced by electronic interlockings, the pace of the upgrades is rather slow, which is mostly due to the very high reliability of relays. However, there are also other technical and economic reasons for the persistence of legacy systems in the railway sector.

On many rail networks, relay interlocking systems have been continuously operating for over 50 years. This decade-long experience has shown that these systems are highly reliable and safe in case of failure. Previous research [4] has shown that the rate of dangerous failures (λ_D_) for an individual railway relay is 1.4∙10^−11^ h^−1^, and it is 1.8∙10^−7^ h^−1^ for an average relay interlocking. This translates to a mean time to hazardous failure (T_D_) of over 8 million years for individual relays and around 634 years for the interlocking system as a whole.

The relays used for railway applications vary from system to system and from application to application. A general classification of these components is given by the International Union of Railways (UIC) as follows [9]:

- Type N relays, which are used in safety related functions. Multiple design features (materials, structure) ensure that their state will always be dropped in case of any failure. Any fault of the relay cannot produce a dangerous situation, as the restrictive response of the system is correlated with the dropped state of the relay.
- Type C relays, which can be used in safety-related functions. They are smaller than type N relays, which translates to a lower production cost due to less resources being needed. The downside of these relays is that their state must be constantly monitored with additional circuitry.
- Non-signal relays, which may not be used for any safety-related functions.

The increased reliability of relay interlockings serves as a deterrence for many network managers to upgrade to the latest technology, especially on branches where the rail traffic is not intensive. From a design point of view, relay interlockings are the standard against which modern electronic interlocking systems are compared to when evaluating their performances [10].

A unique challenge posed by the persistence of these legacy systems is that fewer railway specialists possess a deep and comprehensive understanding of them [10]. This unmet need of qualified personnel may lead to increased waiting times when dealing with disruptions of the interlocking.

To compensate for this vulnerability, support systems may be designed and implemented. Support may be given by simulating relay interlockings to automatically detect possible design flaws or weaknesses. Such simulations are dependent on reliable and accurate interlocking models. The generation of these models usually involves the digitalization of existing relay schematics either in a specially designed schematics software [10] or by recoding them as an XML file [11]. The language used to describe the behavior of relay interlockings is usually Boolean [12] or LLD [13]. Regardless of the generation method, all models are checked using formal methods [4,14,15].

Another type of support systems focuses on easing the maintenance of the already installed interlocking hardware. The main purpose of this kind of systems is to facilitate easier maintenance so that it can be achieved with a lower number of qualified personnel. Great importance is placed on railway relays, as they are the key component of the interlocking. 

Research has gone into the automatic detection of faults inside railway relays during their scheduled maintenance [16]. By recording transient currents and analyzing them by means of artificial neural networks, it is possible to distinguish between multiple types of faults (such as misshapen armatures, coil short-circuit) with a reliable degree of certainty [17].

The use of sensors for support systems in railways has largely been restricted to traffic management applications or to infrastructure monitoring [18,19]. Due to the large covered area, such sensors are usually part of wireless sensor networks (WSN) [20]. Sensor applications for interlocking systems have, to the extent of our research, not been extensively researched.

## 2. Background Information

### 2.1. Basic Concepts 

One of the three elementary passive components, the coil, is built by successive windings of a conductor. The coil core may be constructed of various ferromagnetic materials or may be non-existent (air) [21]. From a functional point of view, the coil has the property of storing energy in the magnetic field [22].

Like many fields in physics, the magnetic field is a vector field. Any point inside it is characterized by a magnitude and direction by the B vector, which is called magnetic induction. Magnetic fields can be easily studied regarding their shapes and the changes produced inside them by different elements (coils, magnets, metal objects, etc.). The B vector is used to represent these magnetic field lines, and the unit of measurement used for magnetic induction is Tesla [23].

For a conductor modeled in the form of a single radius loop with a current I, the magnetic induction along the axis perpendicular to it is calculated as:

(1) $$B_{x}=\frac{\mu_{0}{\mathrm{Ia}}^{2}}{2{\left(x^{2}+a^{2}\right)}^{3/2}},$$

where µ_0_ is the void permeability (4*π ×* 10^−7^).

In the case of a solenoid consisting of N loops of the same size, the magnetic induction along the axis is:

(2) $$B_{x}=\frac{\mu_{0}{\mathrm{NIa}}^{2}}{2{\left(x^{2}+a^{2}\right)}^{3/2}}.$$

The maximum value of the magnetic field is recorded in the center of the solenoid (x = 0), where the induction has a simplified form:

(3) $$B_{x}=\frac{\mu_{0}\mathrm{NI}}{2a}.$$

**Figure 1:**
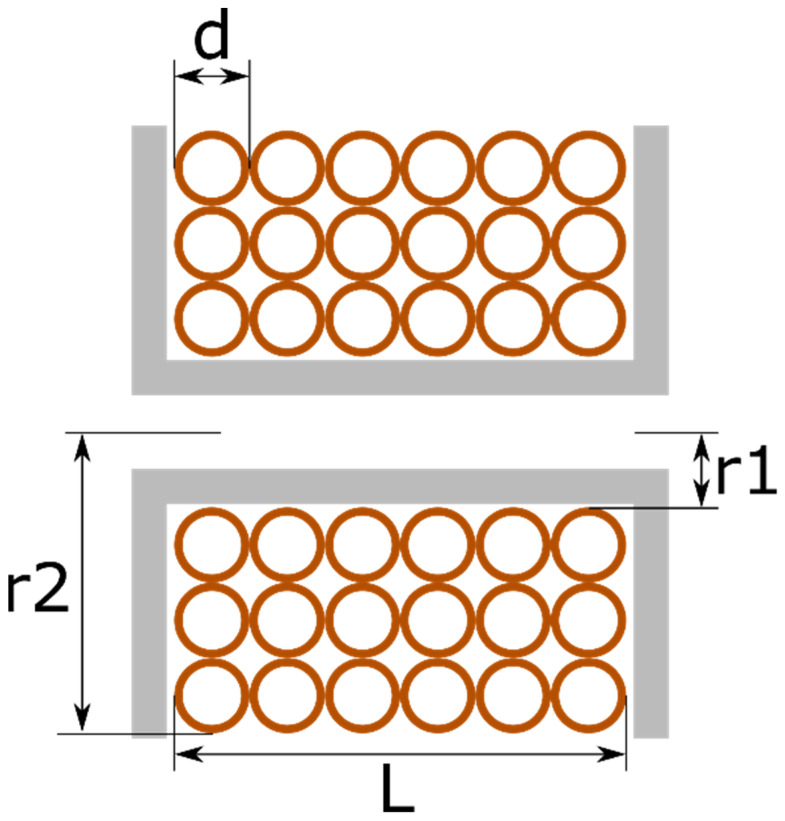
Structure of a multi-layer coil.

For multi-layer coils, the magnetic induction along the axis is modeled by the expression [24]:

(4) $$B_{x}=\frac{\mu_{0}\mathrm{NI}}{2L\left(r_{2}-r_{1}\right)}\left(A\;\ln\frac{r_{2}+\sqrt{r_{2}^{2}+A^{2}}}{r_{1}+\sqrt{r_{1}^{2}+A^{2}}}+B\;\ln\frac{r_{2}+\sqrt{r_{2}^{2}+B^{2}}}{r_{1}+\sqrt{r_{1}^{2}+B^{2}}}\right).$$

where

(5) $$A=\frac{L}{2}-x.$$

(6) $$B=\frac{L}{2}+x$$

and L represents the coil length, r_1_ is the inner radius, and r_2_ is the outer radius (Figure 1).

### 2.2. Simulation of Magnetic Fields with Magpylib

Magpylib is a Python programming language pack designed specifically to simulate magnetic fields generated by various sources. Among them are rectangular, cylindrical or spherical parallelepiped magnets, straight or loop conductors traversed by currents and dipoles [25].

Magnetic field sources can be rotated or moved in space to study the interaction between them. For magnet and dipole sources, the magnetization vector can be defined according to the three spatial components (O*x*, O*y*, O*z*), while for conductors, the current flowing through them can be defined [26].

The measurement units used by the Magpylib package are:

- mT—for magnetic induction (B) and magnetization (µ0M);
- kA/m—for the magnetic field strength (H);
- mm—for dimensions and positions;
- degrees—for angles;
- A—for currents.

**Figure 2:**
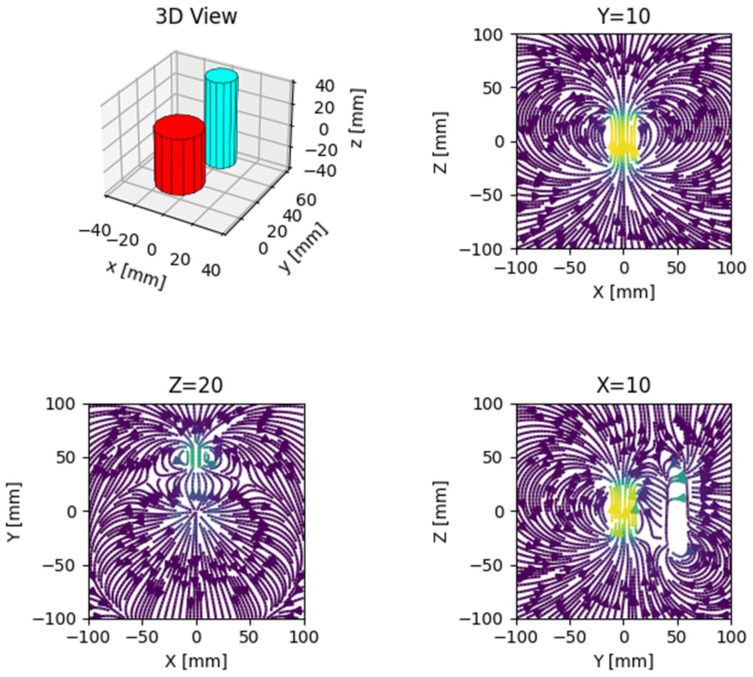
The shape of the magnetic field generated by two magnets observed from different planes.

In addition to defining and manipulating different sources, it is possible to calculate and represent the induction (B) and the intensity of the magnetic field (H). For this, we have used the Magpylib package to calculate and represent the magnetic field generated by two cylindrical magnets (Figure 2).

### 2.3. General Information about the Romanian Rail Relays

Interlocking relays installations in Romania (types CR-2 and CR-3) use three types of electromagnetic relays: N relays, C relays and non-signal relays [27]. Each of these types of relays is used specific applications.

The N relays are the most common ones and are used in the realization of logic circuits for the verification of the safety conditions in the interlocking installation and for the control of field objects (switches, signals, etc.). From a constructive point of view, N relays contain a control circuit consisting of one or two coils and an actuating circuit consisting of a maximum of eight groups of contacts that close and open depending on the supply of the coils [27].

Depending on the number of coils, their resistors and the number of contact groups, there are several types of N relays. In terms of magnetic characteristics, it is important to mention the parameter values for the coils used. Table 1 contains the main parameters of N relays.

**Figure 3:**
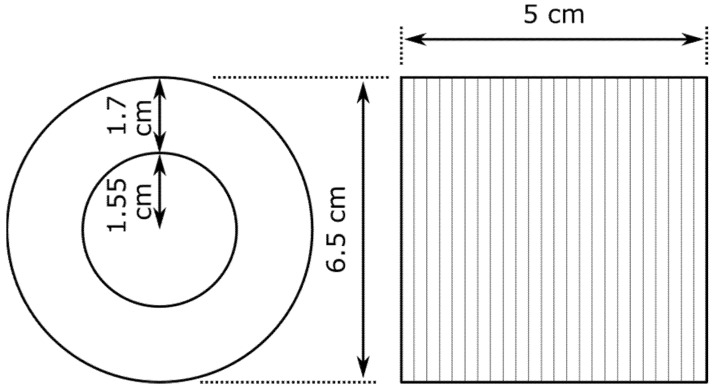
Coil dimensions used in N relays with two coils.

N relays have a standard shape and size, regardless of their type. The entire set of coils and contacts is contained in a box measuring 230 × 80 × 201 mm and weighs approximately 4 kg. The coil sizes for the two-coil N relays are shown in Figure 3.

### 2.4. Modeling of Rail Relays

The behavior of the magnetic field generated by the coils of railway relays can be modeled using the Magpylib package. Taking into account the multi-layer structure of the relay coils, the induction was calculated by entering in Formula (4) the parameters required for computation. As an example, for a single coil of an NF1-800 relay, the parameters have the following values:

- µ_0_ = 4π × 10^−7^;
- N = 8800;
- I = 60 mA (assuming a plug in value of 24 V);
- L = 5 cm;
- r_1_ = 1.55 cm;
- r_2_ = 3.25 cm.

**Figure 4:**
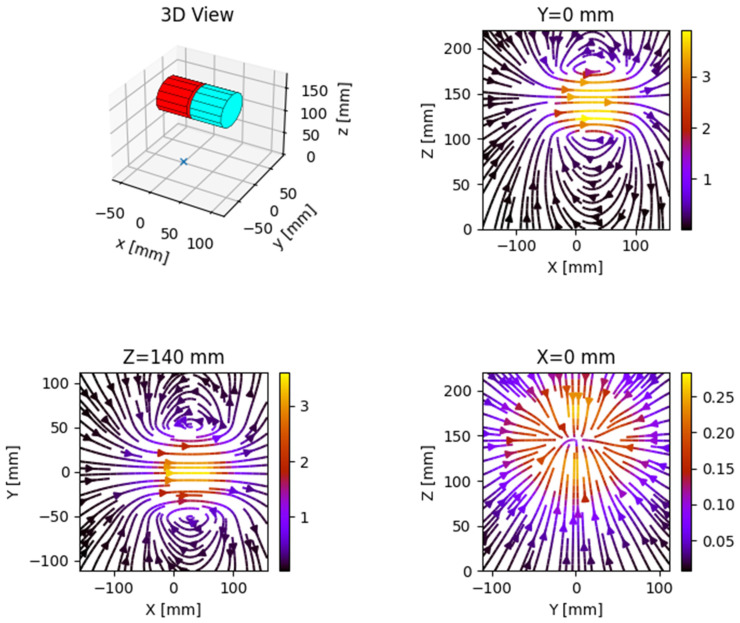
The appearance of the magnetic field simulated with Magpylib.

The calculated e in the center of a coil is 9.61 mT in the case of a single coil being powered and 4.81 mT in the case both coils were powered. Using these parameters, it was possible to simulate the magnetic field generated by the two coils of the relay, as seen in Figure 4. The two coils were arranged similar to their real counterparts on the same axis (Y) with a distance of 5 mm between them and at a height of 145 mm.

## 3. Research Conducted

### 3.1. Research Methodology

In order to determine the shape of the magnetic field around railway relays and the changes that occurred during operation, a spatial distribution of the measurements was needed. The measuring space was defined around a N rail relay, considering the characteristics of the sensor being used.

**Figure 5:**
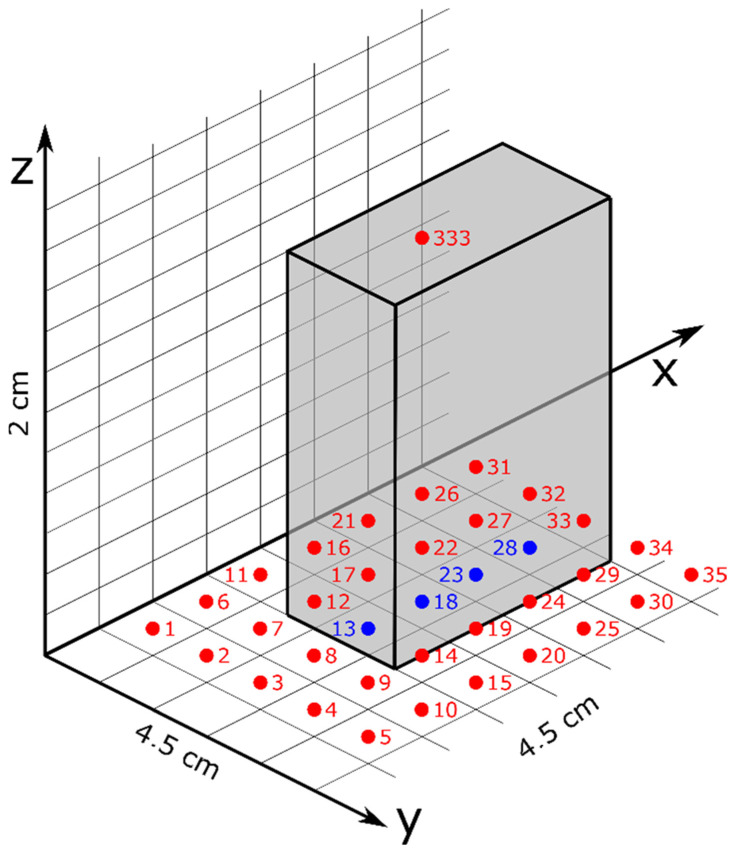
The measurement grid defined around a railway relay (gray) figuring some of the directly measured points (red) and some of the indirectly measured points (blue).

The measurement grid contains 420 individual points (Figure 5) at which the measurements were made. The points were distributed at a distance of 4.5 cm in length and width (*x* and *y* axes) from each other and in height (*z* axis) at a distance of 2 cm. In total, the measurement grid measures 27 cm (*x*-axis), 18 cm (*y*-axis) and 22 cm (*z*-axis), which goes far beyond the dimensions of a railway relay. This allows the evaluation of the influence of the magnetic field produced by a relay on its neighbors.

The relay was placed on the grid so that the longer side of its base was parallel to the O*x* axis and the narrower side was parallel to the O*y* axis. Measurements started at point 1 (Figure 5) and were carried out in sequence by advancing first on the y axis (points 2–5), which was followed by advancements on the *x* axis (points 6–10). After completing all measurements on the first *x*O*y* plane, the next measurement was carried out at the same *x* and *y* coordinates as point 1 but shifted one position upwards on the *z* axis. All subsequent measurements for the other *x*O*y* planes were carried out following the same procedure.

Due to the presence of the relay on the measuring set-up, some points could not be directly measured by the sensor (e.g., points 13, 18, 23, 28). For these positions, the corresponding values were calculated as averages of the neighboring points, which could be directly measured.

The measurement grid and associated housing of the measuring setup were built out of LEGO ^®^ components. This allowed for a flexible environment regarding the positioning of the sensor and offered pre-established standard dimensions for the measuring grid.

The magnetic field components on each axis were saved for each measurement point in a .csv table. The data were subsequently processed and interpreted in order to highlight the main observations.

**Figure 6:**
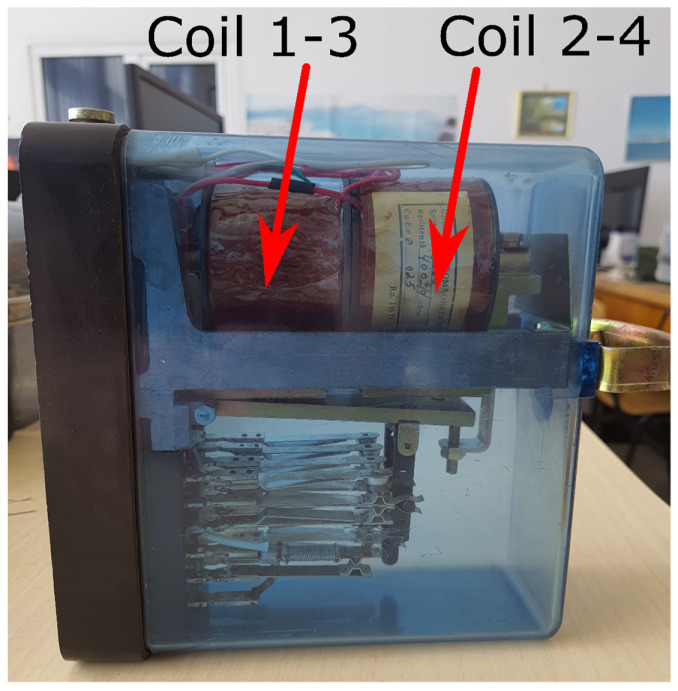
Typical arrangement of coils in an NF-1 800 relay.

The relay used is of NF1-800 type, which is the most used N type relay [27]. Four sets of distinct measurements were performed, corresponding to the following states of the relay: relay not powered at all, picked state with coils 1–3 powered, picked state with coils 2–4 powered, and picked state with both coils powered. Relay coils are presented in Figure 6.

### 3.2. Measuring Setup

**Figure 7:**
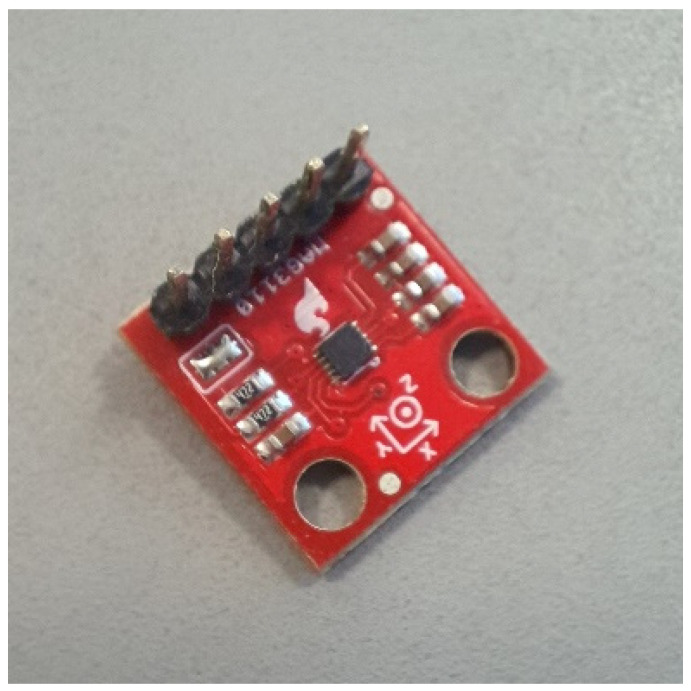
MAG3110 magnetometer.

The system used to measure the magnetic field was designed around the MAG3110 sensor (Figure 7). This is a magnetometer that can measure the intensity of the magnetic field on three axes (O*x*, O*y* and O*z*), and that transmits this information via a I2C serial interface. One of the main applications of the device is to function as an independent electronic compass together with an accelerometer [28,29].

The main parameters mentioned in the data sheet [28] are:

- Supply voltage: 1.95–3.6 V;
- Measuring range: −1000–1000 µT;
- Sensitivity: 0.10 µT/LSB;
- Turn-On Delay Time: 2.5 ns;
- Turn-On Rise Time: 9 ns;
- Turn-Off Delay Time: 20 ns;
- Turn-Off Fall Time: 7 ns.

The data collection via the magnetometer is controlled by an Arduino Nano development board. It communicates via the I2C serial interface with the magnetometer and then transmits the measurements to a computer connected to it via the USB serial interface.

**Figure 8:**
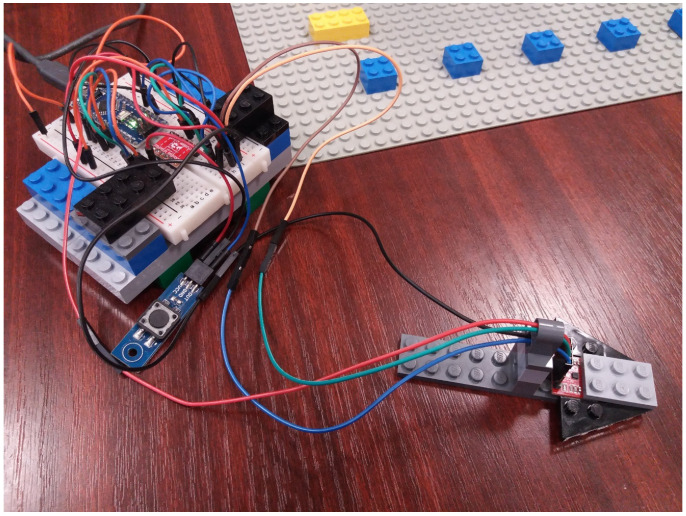
Experimental setup.

Because the magnetometer and the development board operate with different supply voltages, the assembly contains a logic level converter between 3.3 and 5 V. Figure 8 shows the measurement assembly used. The button is used to initiate a measurement.

For each point defined on the measurement grid, three consecutive measurements were automatically performed. The average of these measurements was transmitted through the serial interface to the computer. The data was saved in a “.csv” format via the ArduSpreadsheet extension [30].

### 3.3. Data Processing

The four sets of measurements were first processed using a spreadsheet software to perform arithmetic averages corresponding to the positions where the magnetometer could not be placed.

Based on the data gathered, representations of the shape of the magnetic field in different planes of the measurement grid were prepared. Graphical representations were made in Python.

## 4. Results

**Figure 9:**
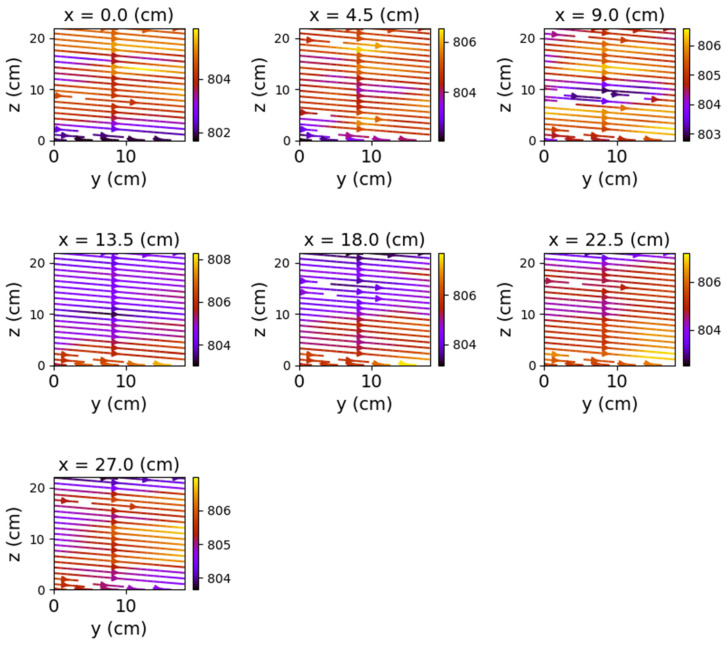
Field lines for *y*O*z* plane—non-powered relay.

**Figure 10:**
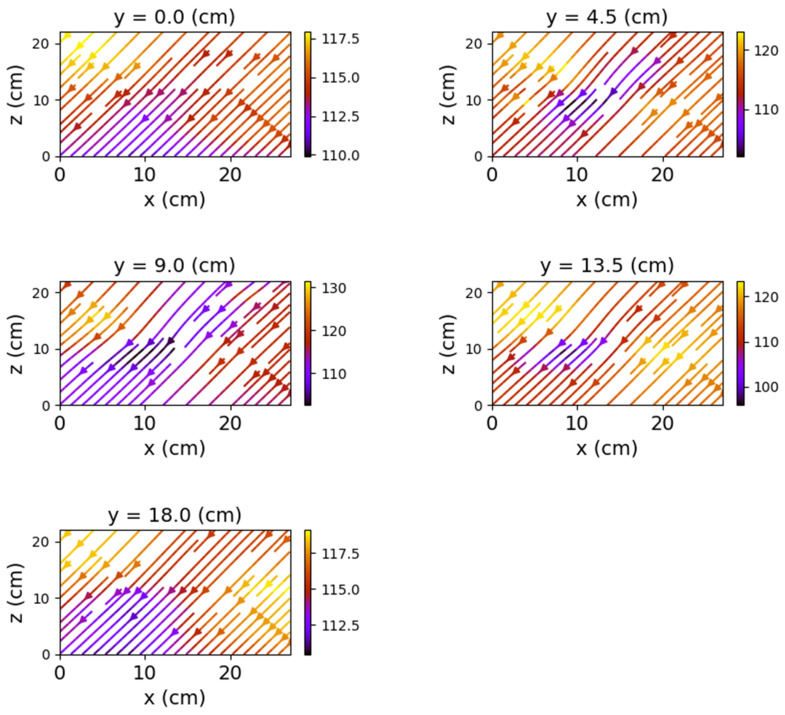
Field lines for *x*O*z* plane—non-powered relay.

**Figure 11:**
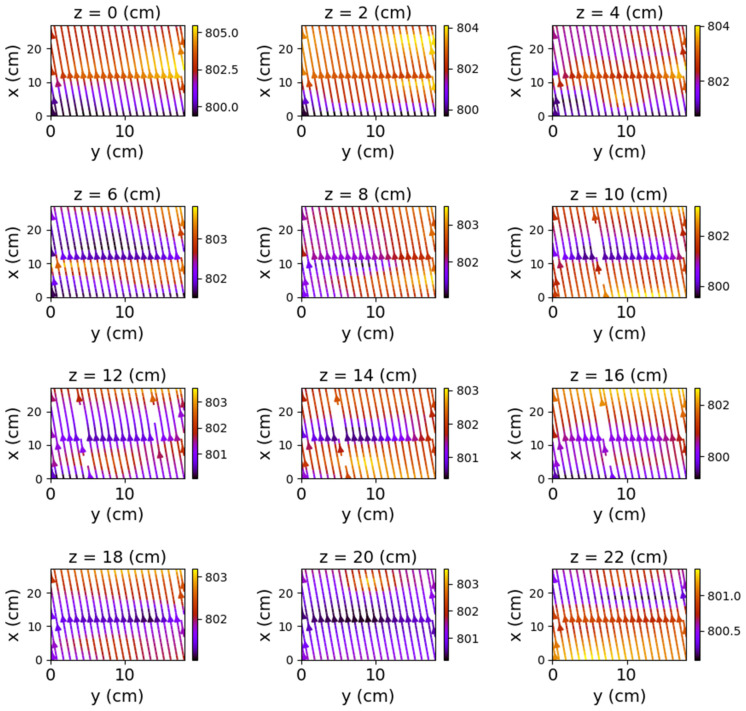
Field lines for *x*O*y* plane—non-powered relay.

The first set of measurements corresponds to the non-powered relay. In this case, the represented field lines are generated exclusively by the magnetic field of planet Earth. Their aspect is almost identical for each position of the represented plans. The deformations that occur correspond to the alteration of the field lines by the metallic components of the relay. Figure 9, Figure 10 and Figure 11 have graphical representations of the field lines.

**Figure 12:**
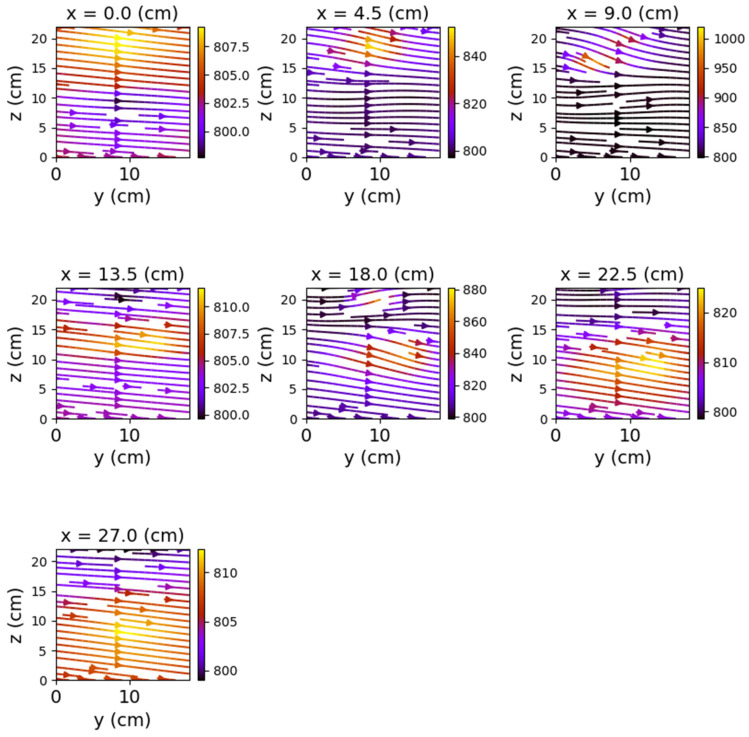
Field lines for *y*O*z* plane—relay with both coils powered.

**Figure 13:**
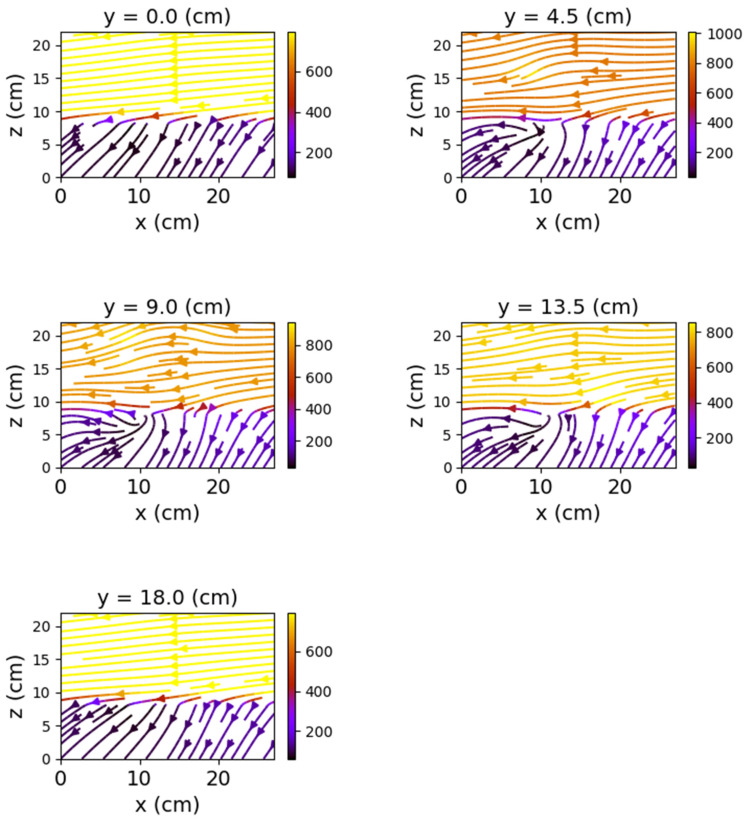
Field lines for *x*O*z* plane—relay with both coils powered.

**Figure 14:**
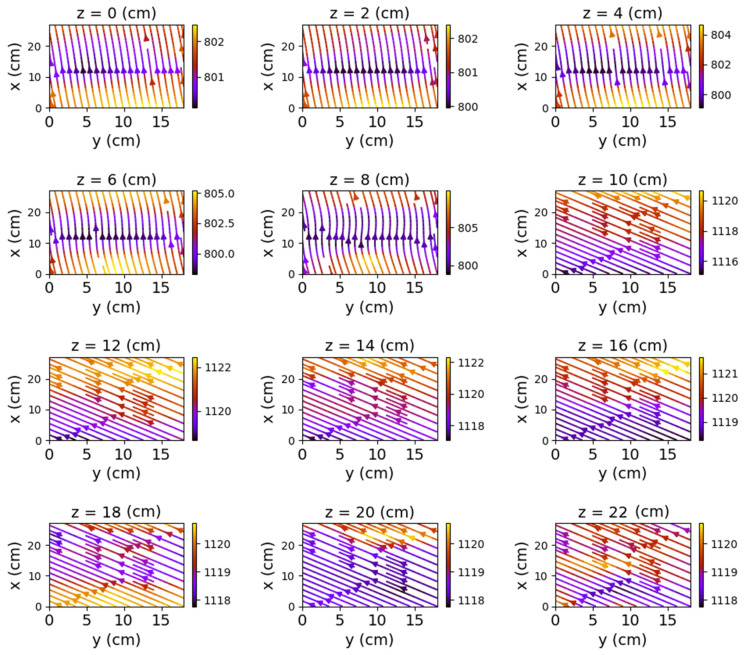
Field lines for *x*O*y* plane—relay with both coils powered.

In the case where both coils were powered, the magnetic field changes significantly, both in intensity and in shape. The differences from the previous scenario are mainly observed in the *y*O*z* and *x*O*z* planes. These are shown in Figure 12, Figure 13 and Figure 14.

Figure 12 shows very clearly the deformation of the field lines as the planes approach the center of the relay. The maximum deformation of the field is recorded at the position *y* = 9 cm, where the *x*O*z* plane intersects the relay, creating two identical halves with respect to the O*x* axis.

Closing the relay using only one of the coils also produced a noticeable change in the field lines. Again, the *y*O*z* and *x*O*z* plans revealed the most obvious changes.

**Figure 15:**
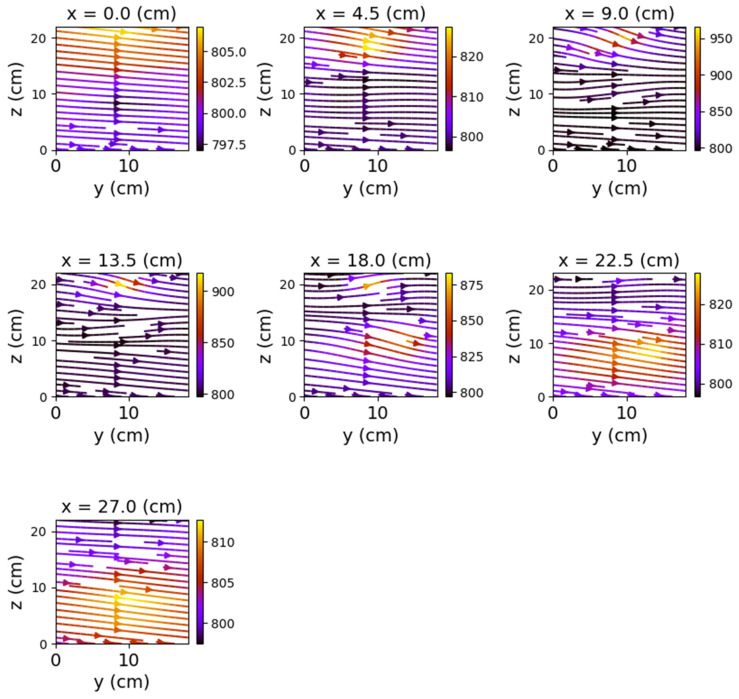
Field lines for *y*O*z* plane—relay with coil 1–3 powered.

**Figure 16:**
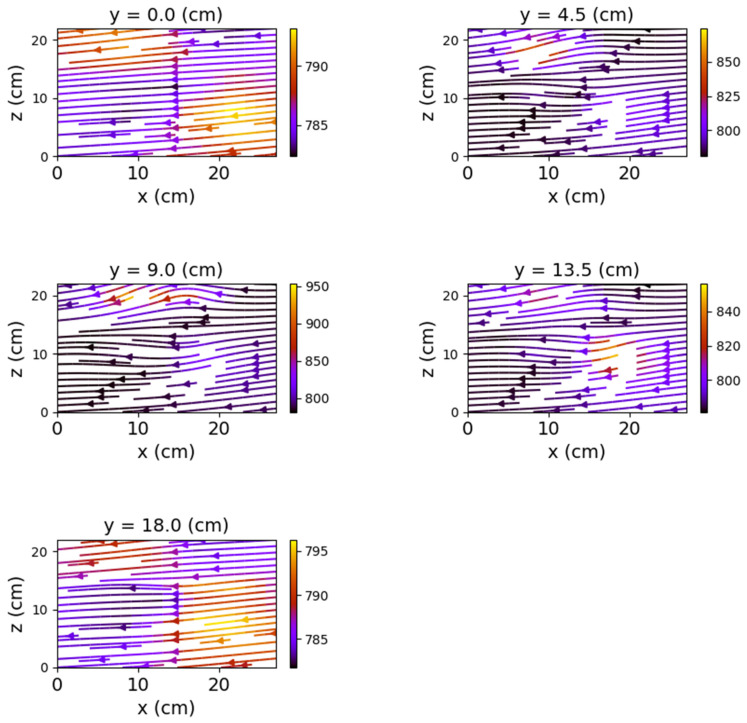
Field lines for *x*O*z* plane—relay with coil 1–3 powered.

**Figure 17:**
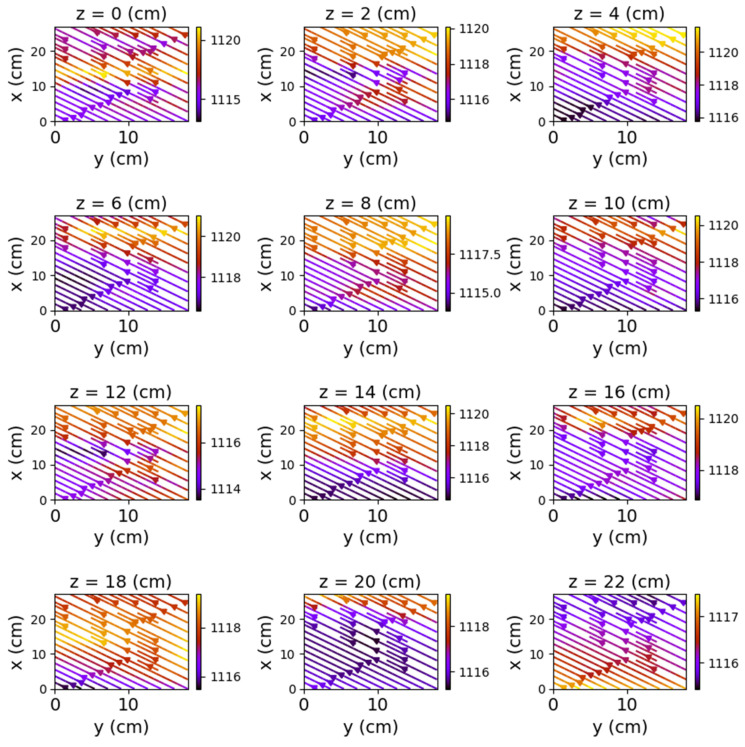
Field lines for *x*O*y* plane—relay with coil 1–3 powered.

**Figure 18:**
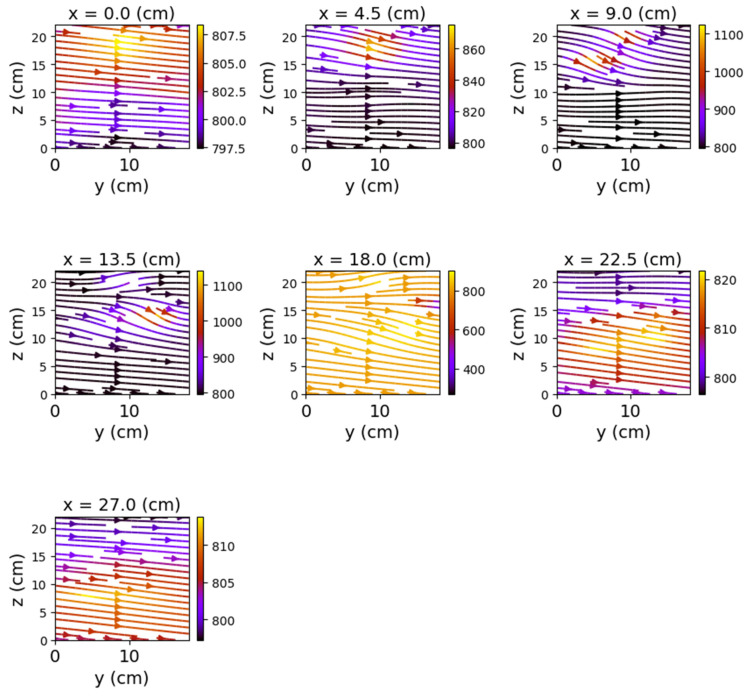
Field lines for *y*O*z* plane—relay with coil 2–4 powered.

**Figure 19:**
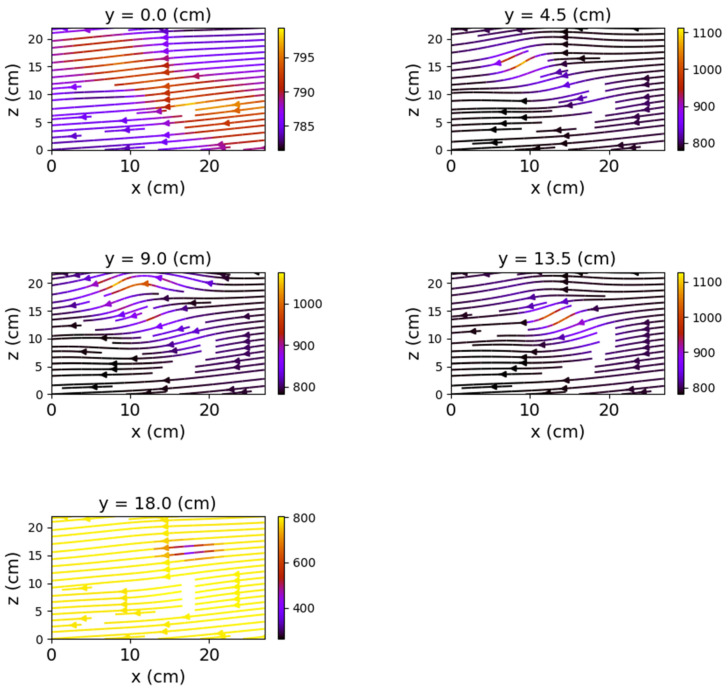
Field lines for *x*O*z* plane—relay with coil 2–4 powered.

**Figure 20:**
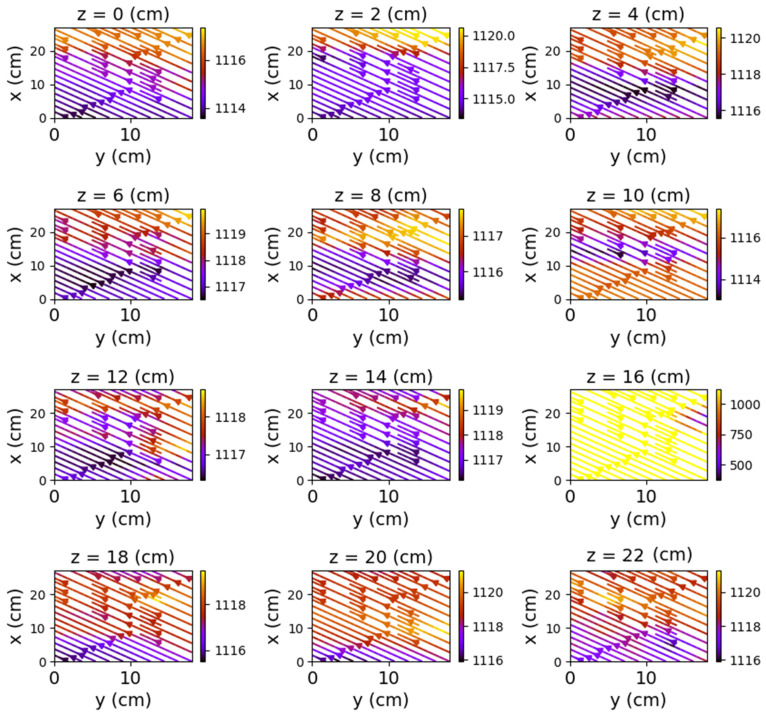
Field lines for *x*O*y* plane—relay with coil 2–4 powered.

Figure 15, Figure 16, Figure 17, Figure 18, Figure 19 and Figure 20 present the results obtained for only one coil powered (1–3 and, respectively, 2–4).

## 5. Discussion

Based on the data presented above, a number of observations can be made regarding the usefulness of magnetometer-type sensors.

**Figure 21:**
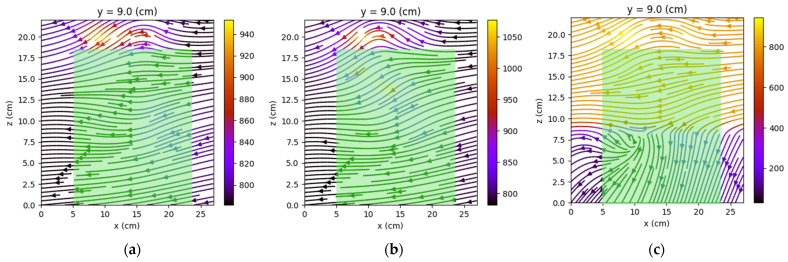
The shape of the field lines for the three possibilities of picked relay: C1-3 (**a**), C2-4 (**b**), and both coils (**c**). The green area represents the measured relay.

The first observation is that the most obvious deformations of the magnetic field were recorded in the *x*O*z* planes. One may observe that the graphical representations also reveal the relay power supply. Figure 21 compares the shape of the field lines for the same *x*O*z* plane in the three possible supply alternatives.

The deformations in the *y*O*z* planes are also noticeable but without being able to notice a substantial difference depending on the supply of the coils. The smallest changes are recorded in the *x*O*y* plane. Therefore, the discrimination between the different states of the relay should be achieved by measuring magnetic induction in the *x*O*z* plane.

Based on the measured deformations of the magnetic field, the optimal positioning of the magnetometer was deduced to be on the relay’s housing, at the top. It should be placed above the transition area between the two coils of the relay. This positioning corresponds very closely to point 333 on the measuring grid (Figure 5) and allows for the accurate detection of the picked state regardless of the powering scenario of the relay coils. Table 2 contains the values measured on the three axes in the recommended mounting position as well as the calculated induction magnitude of the three planes based on them.

To correctly determine the state of the relay at the recommended location of the sensor, it is recommended to monitor the induction magnitude on the *x*O*z* and *x*O*y* planes. For the former, an increase of over 600 µT relative to the dropped state is to be interpreted as a change in the state of the relay, while for the latter, an increase of 200 µT is to be interpreted as such. This corresponds to the earlier observations regarding the observed deformation of the magnetic field being most noticeable on the *x*O*z* plane.

From the shape of the magnetic field in the *y*O*z* and *x*O*z* planes, it can be noticed that the influence of the powered coils decreases rapidly outside the relay. Thus, their influences on neighboring relays can be neglected.

The measurements carried out for the non-powered relay illustrate the influence of the geomagnetic field on the sensor. Its magnitude varies between 27 µT in South America to around 61 µT in Siberia [31]. This does not pose any challenge in detecting the state of the relay, as the measured magnitudes for the three powered cases are significantly different from the base scenario.

The influence of other railway equipment such as track circuits, axle counters or of the railway supply system is negligible as the relay racks are placed in a separate room with enough physical separation to prevent such interference.

## 6. Conclusions

The evaluation of the relay involved the design of a measurement circuit and its use to gather data related to the magnetic field around the railway relays in several operating modes. The modeling of the magnetic fields had the purpose to identify the optimal location of the magnetometer on the surface of the relay. This was found to be on top of the relay, centered. In this position, the sensor is able to accurately determine the state of the relay. Furthermore, a comparison system will be able to evaluate if the state is corresponding to the command given (coils powered) and trigger an alert, if necessary. In the placement proposed in this paper, the value obtained for powered relay was 6.9–7.2 times higher on the *x*O*z* plane compared to non-powered state, making it easily detectable. 

In this study, accuracy was also taken into consideration, as more measurements were performed for each state, as presented in the paper. From the values gathered, we were able to determine that the standard deviation for the measurement set was:

- 0.37 for *x* data;
- 0.36 for *y* data; and
- 0.30 for *z* data.

This proves that there are not any spikes in the data collected and, considering that the values obtained for powered relay are far away from the non-powered state, a false response of such system has a very small probability of occurrence.

The influence on neighboring relays could also be assessed. This was determined to be very low, which avoids the occurrence of the false response of the magnetometer in the case of powering the neighboring relays. Therefore, a monitoring system based on magnetic sensors proved to be a cheap solution to further maintain existing relay interlocking systems, thus eliminating the expensive solution of total system replacement.

Because relay interlockings use different types of relays with their own parameters, each potential application that uses magnetic sensors needs to be properly calibrated to ensure a correct answer. The development of accurate relay simulation models is crucial to this endeavor. In this paper, we built such a model using the Magpylib Python package. The results of the simulations indicate that the model is accurate to a certain degree with the physical measurements of the relay. The model needs to be further improved in order to correctly represent the influence other components (e.g., metallic components) have on the magnetic field. 

The measurements presented in this paper were carried out exclusively on DC relays. The described application of magnetic sensors may also be implemented for AC relays. Further research is necessary to establish whether and what changes would be required for this purpose.

## Figures and Tables

**Table 1:** Main characteristics of N relays.

Relay Type	Conductor Diameter [mm]	No of Turns	Coil Resistance [Ω]	Coil Connection
NF1-800	0.23	2 × 8800	2 × 400	Series
NF1L-400	0.25	2 × 4500	2 × 200	Series
NF1L-200/400	0.25/0.23	6500/8800	200/400	Independent
NF1L-200/30	0.25/0.47	6500/2600	200/30	Independent
NF1-2	1.25	1000	2	Independent
NF2-2	1.25	1000	2	Independent
NF2-800	0.23	17,600	800	Independent
NF1-2000	0.21	2 × 15,800	2 × 1000	Series
NF2-40	0.55	2 × 2200	2 × 20	Series
NF1T-800	0.23	2 × 8800	2 × 400	Series
NIF1-150	0.27	2 × 8100	2 × 300	Parallel

**Table 2:** Induction (µT) determined for measuring point 333.

Relay State	*x*	*y*	*z*	||*x*O*z*||	||*y*O*z*||	||*x*O*y*||
Not powered	−57.99	799.2	−96.59	112.661	805.016	801.301
Coil 1–3 powered	−783.28	798.765	−193.605	806.852	821.893	1118.728
Coil 2–4 powered	−782.34	797.84	25.46	782.754	798.246	1117.410
Both coils powered	−783.38	798.86	−78.83	787.336	802.740	1118.866

## Data Availability

Not applicable.

## References

[1] Bešinović N. (2020). Resilience in railway transport systems: A literature review and research agenda. Transp. Rev..

[2] Huang L. The Past, Present and Future of Railway Interlocking System. Proceedings of the 2020 IEEE 5th International Conference on Intelligent Transportation Engineering (ICITE).

[3] Preuß E. (2012). Stellwerke Deutscher Eisenbahnen Seit 1870.

[4] Anders E., Berndt T., Dolgij I., Ivančenko V., Lykov A., Márton P., Maschek U., Mongardi G., Nasedkin O., Nikitin A. (2018). Railway Signalling & Interlocking: International Compendium.

[5] Efanov D., Lykov A., Osadchy G. Testing of relay-contact circuits of railway signalling and interlocking. Proceedings of the 2017 IEEE East-West Design & Test Symposium (EWDTS).

[6] Pachl J. (2021). Railway Signalling Principles.

[7] Pachl J. (2018). Railway Operation and Control.

[8] Bäckman R., Oliver I., Limonta G. Integrity Checking of Railway Interlocking Firmware. Proceedings of the Computer Safety, Reliability, and Security. SAFECOMP 2020 Workshops.

[9] International Union of Railways (2018). Use of Signalling Relays.

[10] Amendola A., Becchi A., Cavada R., Cimatti A., Ferrando A., Pilati L., Scaglione G., Tacchella A., Zamboni M. (2022). NORMA: A tool for the analysis of Relay-based Railway Interlocking Systems. Lecture Notes in Computer Science, Proceedings of the Tools and Algorithms for the Construction and Analysis of Systems. TACAS 2022, Munich, Germany, 2–7 April 2022.

[11] Haxthausen A.E., Kjær A.A., Le Bliguet M. (2011). Formal Development of a Tool for Automated Modelling and Verification of Relay Interlocking Systems. Lecture Notes in Computer Science, Proceedings of the FM 2011: Formal Methods, Limerick, Ireland, 20–24 June 2011.

[12] Martinez S., Pereira D.I.D.A., Bon P., Dutilleul S.C., Perin M. (2020). Towards safe and secure computer based railway interlocking systems. Int. J. Transp. Dev. Integr..

[13] Bonacchi A., Fantechi A., Bacherini S., Tempestini M., Cipriani L. (2013). Validation of Railway Interlocking Systems by Formal Verification, A Case Study. Lecture Notes in Computer Science, Proceedings of the Software Engineering and Formal Methods, SEFM 2013, Madrid, Spain, 23–27 September 2013.

[14] Busard S., Cappart Q., Limbrée C., Pecheur C., Schaus P. (2015). Verification of railway interlocking systems. Proceedings 4th International Workshop on Engineering Safety and Security Systems, ESSS 2015, Oslo, Norway, 22 June 2015.

[15] Limbree C., Cappart Q., Pecheur C., Tonetta S. (2016). Verification of railway interlocking—Compositional approach with OCRA. Reliability, Safety, and Security of Railway Systems. Modelling, Analysis, Verification, and Certification. First International Conference, RSSRail 2016, Paris, France, 28–30 June 2016, Proceedings.

[16] Havryliuk V. The Wavelet Based Detecting of the Signalling Relay Armature Defects. Proceedings of the 2019 IEEE 2nd Ukraine Conference on Electrical and Computer Engineering (UKRCON).

[17] Havryliuk V. (2017). Fault Diagnosis for Electromagnetic Relay Using Discrete Wavelet Transform and Wavelet Packet Entropy.

[18] Efanov D.V., Osadchy G.V., Khoroshev V.V. Testing of Optical Sensors in Measuring Systems on Railway Marshalling Yard. Proceedings of the 2018 IEEE East-West Design & Test Symposium (EWDTS).

[19] Lee S.J., Ahn D., You I., Yoo D.Y., Kang Y.S. (2020). Wireless cement-based sensor for self-monitoring of railway concrete infrastructures. Autom. Constr..

[20] Iwasawa N., Kawamura T., Iwaki N., Ryuo S., Nozue M. (2019). Design of Wireless Sensor Network in the Railway Facilities. Communications in Computer and Information Science.

[21] Cormoș A.C., Iordache V., Surugiu C.M. (2011). Materiale și Componente Pasive Electronice—Noțiuni Fundamentale.

[22] ElectronicsTutorials, “The Inductor,” [Interactive]. https://www.electronics-tutorials.ws/inductor/inductor.html.

[23] Young H.D., Freedman R.A. (2012). Sears and Zemansky’s University Physics with Modern Physics.

[24] Basharat M., Ding M., Cai H., Li Y., Fang J. (2017). Design and Analysis of Multilayer Solenoid Coil for Faraday Modulator. MATEC Web of Conferences.

[25] Ortner M., Bandeira L.G.C. (2020). Magpylib: A free Python package for magnetic field computation. SoftwareX.

[26] Ortner M. Magpylib Documentation Release 3.0.2, [Interactive]. https://magpylib.readthedocs.io/_/downloads/en/stable/pdf/.

[27] Stan A.I., David S. (1983). Centralizări Electrodinamice și Bloc de Linie Automat.

[28] Freescale Semiconductor (2013). Xtrinsic MAG3110 Three-Axis, Digital Magnetometer. MAG3110 Rev. 9.2. NXP. https://www.nxp.com/docs/en/data-sheet/MAG3110.pdf.

[29] Sparkfun “SparkFun Triple Axis Magnetometer Breakout—MAG3110, [Interactive]. https://www.sparkfun.com/products/retired/12670.

[30] Luuk I. Logging Arduino Serial Output to CSV/Excel (Windows/Mac/Linux), [Interactive]. https://circuitjournal.com/arduino-serial-to-spreadsheet.

[31] Centers N. World Magnetic Model—Maps of Magnetic Elements, Noaa.gov, Published 2020. https://www.ngdc.noaa.gov/geomag/WMM/image.shtml.

